# Current Status of Familial Hypercholesterolemia in China: A Need for Patient FH Registry Systems

**DOI:** 10.3389/fphys.2019.00280

**Published:** 2019-03-20

**Authors:** Peipei Chen, Xi Chen, Shuyang Zhang

**Affiliations:** Department of Cardiology, Peking Union Medical College Hospital, Chinese Academy of Medical Sciences and Peking Union Medical College, Beijing, China

**Keywords:** familial hypercholesterolemia, cardiovascular disease, FH registry system, cascade screening, dyslipidemia

## Abstract

**Background:** Familial hypercholesterolemia (FH) greatly facilitates the development of cardiovascular disease (CVD). Without timely treatment, the incidence of coronary heart disease (CHD) in patients with FH is 3 to 4 times that in non-FH patients, and the onset of CVD would be advanced by approximately 10 years. There is ample evidence that the diagnosis and adequate treatment of FH are not properly considered for all ethnicities. The monogenic cause of FH includes apolipoprotein B (APOB), low-density lipoprotein receptor (LDLR), and proprotein convertase subtilisin/kexin 9 (PCSK9). There are approximately 2,765,420 to 6,913,550 cases of potential heterozygous FH (HeFH) and 2,205 to 4,609 cases of potential homozygous FH (HoFH) in China. Nevertheless, China lacks clinical diagnostic criteria specific to Chinese patients, such that most FH patients cannot be diagnosed until middle age or after their first cardiovascular event, thus precluding early treatment.

**Objective:** This article explores the gene mutations, diagnosis and treatment of FH patients in China. Following the implementation of the two-child policy, there is a need to establish Chinese FH registry systems and genetic databases and to address the challenges in conducting cascade screening and long-term management.

**Conclusion:** Advocating the establishment of FH registry systems and databases is an important rate-limiting step in improving long-term prognosis in FH patients, so that joint efforts of clinical experts and public communities are required. We recommend a process flow from case identification to entry into the registry system, and the widespread use of the system in clinical applications can provide the best treatment guidance for medical practice.

## Familial Hypercholesterolemia (FH)

Familial Hypercholesterolemia (OMIM#143890) individuals exhibit high serum cholesterol and low-density lipoprotein-cholesterol (LDL-C) levels, the majority of them with tendon xanthomas. Without timely treatment, the incidence of coronary heart disease (CHD) in FH patients is 3 to 4 times that of non-FH patients, and the onset of cardiovascular disease (CVD) would be advanced by approximately 10 years (Huijgen et al., 2012b). The mode of FH inheritance is primarily autosomal dominant (AD) inheritance, followed by autosomal recessive (AR) inheritance. The associated AD genes include *LDLR* (Brown and Goldstein, 1974), apolipoprotein B (*APOB*) (Innerarity et al., 1987), and proprotein convertase subtilisin kexin-9 (*PCSK9*) (Abifadel et al., 2003). Mutations in the LDLR adaptor protein 1 (*LDLRAP1*) gene cause an AR form of FH (Garcia et al., 2001). While a mutation in one allele of the abovementioned genes is likely to cause heterozygous FH (HeFH) phenotypes, simultaneous mutation of two alleles will cause lipid metabolism disorder, a more severe homozygous FH (HoFH) phenotype. If two alleles of the genes of AR form are affected, the patient will present a phenotype that closely resembles HoFH, however, exhibiting a lower plasma LDL-C levels than in HoFH (Fellin et al., 2015).

The World Health Organization has shown that the prevalence of high total cholesterol (TC) has increased noticeably with increases in the income level of a country. In general, the mean TC level in most Asian countries is low compared to other countries in the world (World Health Organization, 2018b); for example, the age-specific and age-standardized mean level of TC in the Chinese population (4.2–4.6 mmol/l) (Tomlinson et al., 2019) is much lower than that in the United States population (5.1–5.7 mmol/l) (Carroll et al., 2015). This difference is related to a low habitual dietary intake of fat and cholesterol in China, and may be the main reason for the low CHD mortality rate in China (He et al., 2004). In previous decades, HeFH has largely been unrecognized in China because patients with HeFH exhibit a lower level of both TC and LDL-C than HeFH patients in other countries (Pimstone et al., 1998); in addition, these subjects did not manifest the usual clinical features (Sun et al., 1994). Several studies have shown the mean TC level (8.9 ± 1.8 mmol/L) and LDL-C value (6.9 ± 1.7 mmol/L) of HeFH patients (Chiou and Charng, 2016) and the mean TC level (18.2 ± 3.9 mmol/L) and LDL-C value (15.9 ± 3.7 mmol/L) of HoFH patients from China (Ma et al., 2018). Another study showed that the plasma TC and LDL-C levels were 9.1 ± 1.5 mmol/L and 7.2 ± 1.5 mmol/L, respectively, in 252 HeFH Chinese patients (mean age 37 ± 17 years, 100 males) (Hu et al., 2013), which are lower compared with the United States definite FH subjects (TC value 10.9 ± 0.49 mmol/L and LDL-C value 9.96 ± 0.04 mmol/L) (Banach et al., 2018). The lower TC and LDL-C values in Chinese FH patients may be partly due to the lower basal TC and LDL-C level in China than in Western countries, mostly because of dietary habits, such as vegetable intake. However, there is no relevant research that clearly indicates whether the difference in basal TC and LDL-C level is related to ethnicity-specific genes.

The genetic burden of many diseases is a fairly major issue in China, as it is a large populous country. Therefore, it is vital to identify characteristics of FH in order to provide an early diagnosis, timely intervention and long-term monitoring of the Chinese FH population.

## Epidemiological Genetics of FH

**FIGURE 1:**
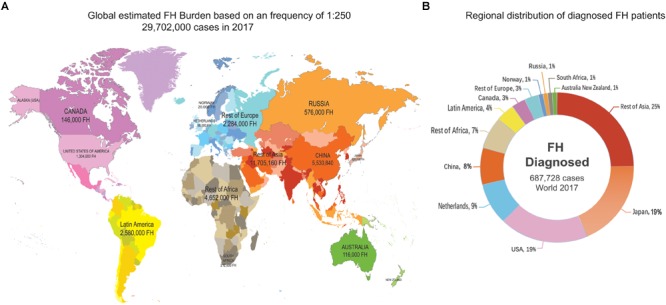
Global FH burden based on a 1:250 frequency and regional distribution of diagnosed FH patients. The source of data was a figure from a previous study (Nordestgaard and Benn, 2017). **(A)** There were global estimated 29,702,000 FH cases in 2017. **(B)** Regional distribution of shows that the total number of confirmed FH patients is 687,728, more than a quarter of them was in Asia.

**FIGURE 2:**
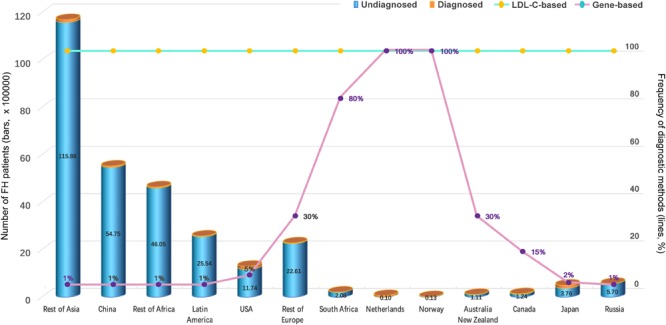
Number of FH Patients and Percentage of Diagnostic Methods (Nordestgaard and Benn, 2017). On LDL-C: the estimated proportion of all FH patients who were diagnosed solely based on LDL-C (probably accompanied by clinical signs of FH). On genes: the estimated proportion of all FH patients who were diagnosed on the basis of the combination of LDL-C, genetic screening of mutations in LDLR, APOB, or PCSK9 genes and possible clinical signs.

Several recent studies have proven the universality of FH, which affects 80% of countries worldwide (Abul-Husn et al., 2016; Benn et al., 2016; Wald et al., 2016). In addition, compared to other genetic diseases, FH happens most commonly in children (Wiegman et al., 2015). It is estimated that a total of 29,702,000 individuals suffer from FH worldwide, but the reported number of FH cases is only 687,728 based on the current estimate of the FH frequency of 1:250 for most countries in which communities were sampled in 2017 (Nordestgaard and Benn, 2017; Figure 1A,B). The estimated number of all diagnosed FH patients is based only on the measurement of LDL-C (probably accompanied by clinical signs of FH) or the combination of LDL-C, genetic screening for mutations and possible clinical signs. While the number of undiagnosed patients accounts for more than 80% of the population of most countries, genetic testing in the Netherlands and Norway is performed in nearly 100% of cases (Figure 2), leading to an improved rate of diagnosis.

**FIGURE 3:**
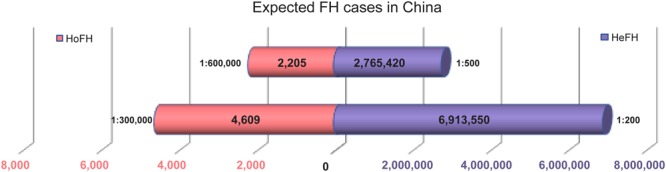
Estimated prevalence of FH in China. Expected HeFH cases and HoFH cases.

China may bear a heavy health burden as the most populous country in the world, accounting for 8% of all FH patients in the world. There has not been a complete investigation of the genetic epidemiology of FH frequency in China so far. According to the 2016 population census conducted in Oct. 2017^1^, it is predicted that there will be 2,765,420–6,913,550 HeFH cases, which is in line with a prevalence rate of 1:200 to 1:500; in addition, it is predicted that there will be 2,205 to 4,609 HoFH cases in line with a prevalence rate of 1:300,000 to 1:600,000 (Figure 3). The International Collaborative Study of Cardiovascular Disease in Asia (InterASIA) China study conducted from 2000 to 2001 showed that the age-standardized prevalence of dyslipidemia was 53.6% in 15,540 Chinese adults 35 to 74 years of age (Gu et al., 2005). Cao et al. reported that FH is truly a common cause of coronary artery disease (CAD) in very young patients (≤ 35 years), with 38.1% of causative mutations detected in China, and the best LDL-C threshold for predicting mutations was 4.56 mmol/L (Cao et al., 2018). It is estimated that there are 2,205–4,609 HoFH patients in China (Figure 3), but only 111 cases with detailed clinical information have been reported so far (Ma et al., 2018). Most of the FH patients in mainland China are undiagnosed and untreated because there remain significant gaps in the awareness of FH among physicians and the general public in China compared with those in other Asian countries (Pang et al., 2015), and the limited data on the prevalence of FH can be due to insufficient genetic screening and national registration.

## Clinical Characteristics, Laboratory Diagnosis and Diagnostic Criteria

Sole reliance on an isolated biochemical screening test is not sufficient to verify the presence of FH, as the cholesterol level in the blood varies with age, race, use of specific drugs, and pathological and physiological conditions; these tests often lead to a false-negative diagnosis or false-positive diagnosis (Henderson et al., 2016). Currently, at least 4 diagnostic systems are widely used for FH diagnosis, namely, the Dutch Lipid Clinic Network (DLCN) (Nordestgaard et al., 2013), the Simon Broome Register (SBR) Group (United Kingdom) (Simon Broome Register Group, 1991), the United States Make Early Diagnosis to Prevent Early Deaths (MEDPED) criteria, and the European Atherosclerosis Society (EAS) (Bruckert, 2014). The abovementioned diagnostic criteria are mainly dependent on a very high TC or LDL-C level in repeat measurements. The most widely used system is the SBR standard, which is simple, effective, economically viable and suitable for routine diagnosis and can be simultaneously used with traditional indicators, medical history and lipid analysis to diagnose FH. Diagnosed or suspected FH can be further confirmed by molecular testing. Of course, the SBR standard has some shortcomings; for example, a large proportion of potential FH patients, mainly patients with mild clinical features and pediatric FH patients without clinical manifestations, may be missed in the analysis. The DLCN standard introduces a scoring system that fully evaluates family history (hyperlipidemia or heart disease) and clinical features (xanthoma, LDL-C elevation, gene mutations), and a diagnosis of FH can be confirmed once a gene mutation is identified. However, patients with gene mutations (8 points) must meet at least one other diagnostic criteria to be diagnosed (greater than 8 points) (Table 1). The SBR and DLCN criteria both take into consideration the molecular diagnostic tests, which are expensive in China. Moreover, lacking resources also prevent many Chinese grassroots hospitals from conducting these tests.

**Table 1:** Dutch lipid clinic network (DLCN) clinical criteria and modified DLCN definition for familial hypercholesterolemia.

DLCN Criteria	Points	Modified DLCN for China	Points
**Family history**		**Family history**	

First-degree relative with known premature (men: <55 years; women: <60 years) coronary artery disease or vascular disease, or first-degree relative with known LDL-C level above the 95th percentile by age, gender for country	**1**	First-degree relative with known premature (men: <55 years; women: <60 years) coronary artery disease or vascular disease.	**1**
First-degree relative with tendinous xanthomata and/or arcus cornealis, or children aged less than 18 years with LDL-C level above the 95th percentile by age, gender for country	**2**		

**Clinical history**		**Clinical history**	

Patient with premature (men: <55 years; women: <60 years) coronary artery disease	**2**	Patient with premature (men: <55 years; women: <60 years) coronary artery disease	**2**
Patient with premature (men: <55 years; women: <60 years) cerebrovascular or peripheral vascular disease	**1**	Patient with premature (men: <55 years; women: <60 years) cerebrovascular or peripheral vascular disease	**1**

**Physical examination**			

Tendinous xanthomata	**6**		
Arcus cornealis prior to age 45 years	**4**		

**LDL-C levels**		**LDL-C levels**	

LDL-C ≥ 8.5 mmol/l (∼330 mg/dl)	**8**	LDL-C ≥ 6 mmol/l (∼230 mg/dl)	**8**
LDL-C 6.5–8.4 mmol/l (∼250–329 mg/dl)	**5**	LDL-C 5.0–5.9 mmol/l (∼190–29 mg/dl)	**5**
LDL-C 5.0–6.4 mmol/l (∼190–249 mg/dl)	**3**	LDL-C 3.5–4.9 mmol/l (∼135–189 mg/dl)	**3**
LDL-C 4.0–4.9 mmol/l (∼155–189 mg/dl)	**1**	LDL-C 2.5–3.4 mmol/l (∼97–134 mg/dl)	**1**

**DNA analysis**			

Causative mutation in the LDLR, ApoB or PCSK9 gene	**8**		
**>8 points Definite FH**			
**6–8 points Probable FH**			
**3–5 points Possible FH**			
**0–2 points Unlikely FH**			

Chinese researchers (Shi et al., 2014) have also improved the modified DLCN standard for Chinese people based on the lower LDL-C baseline levels of Chinese FH patients compared to FH patients in other countries. For the modified DLCN definition, a lower LDL-C distribution for the Chinese population replaced the LDL-C cutoff of the original DLCN criteria for a similar score (Table 1). A recently published paper proposed that 25 (62.5%) and 17 (42.5%) CAD Chinese patients with FH-related pathogenic mutations were undiagnosed according to SBR and DLCN criteria (Cao et al., 2018). It is likely that some Chinese FH patients with TC levels lower than the criteria used in Western countries for identification of potential probands and no clinical manifestations may not be clinically identified.

Previously, China has lacked clinical diagnostic criteria for FH; hence, the diagnosis and treatment rates in the clinic are less than 1% (Vallejo-Vaz et al., 2015). Most FH patients cannot be diagnosed until middle age or after their first cardiovascular event, thus precluding early treatment. Therefore, China needs to develop its own FH diagnostic criteria and registry system based on Chinese characteristics. Recently, the FH consensus in China has been formulated (Department of Cardiovascular Diseases, 2018). Chinese scholars suggest that the combination of gene sequencing and improved DLCN criteria can improve the diagnostic rate of FH in people with premature myocardial infarction. The clinical criteria developed by China included TC/LDL-C level and patients or family members with tendon xanthoma, which were derived from the textbook called Clinical Coronary Heart Disease (Xiao et al., 2014). Selective screening for key populations is recommended. Once FH patients are found, cascade screening of first-degree relatives should be conducted as soon as possible. FH can be diagnosed in adults who meet two of the following criteria: (1) Not treated with lipid-lowering drugs and have serum LDL-C levels ≥4.7 mmol/L; (2) Xanthoma tendinosum or <45 years old with corneal arcus; and (3) FH or premature ASCVD in first-degree relatives, especially in patients with CHD. Diagnostic criteria for FH in children are as follows: untreated blood LDL-C levels ≥3.6 mmol/L, and first-degree relatives with FH or premature CHD. However, China’s stratified medical care exhibits large regional differences and narrow coverage, and the effect of long-term application of the improved standard also requires further observation and analysis.

## Genetic Testing and Cascade Screening

Brown and Goldstein revealed the causes of FH and the way to genetically diagnose FH in 1985, thus wining the Nobel Prize. Genetic testing enhances the sensitivity of FH screening and confirms the potential genotypes of suspected FH populations, making this test of great significance for the establishment of an FH registration system. Genetic testing to determine FH shall be performed by a lipid expert. Once a causative mutation is identified, cascade screening will be performed for the proband and familial relatives (Franke and Lansberg, 2009; Datta et al., 2010). Every new FH case will then become the proband of a much broader cascade (Safarova et al., 2016; Wald et al., 2016). The diagnostic yield of screening among Chinese patients was 2.8 new cases of FH per index case (Wu et al., 2017).

**FIGURE 4:**
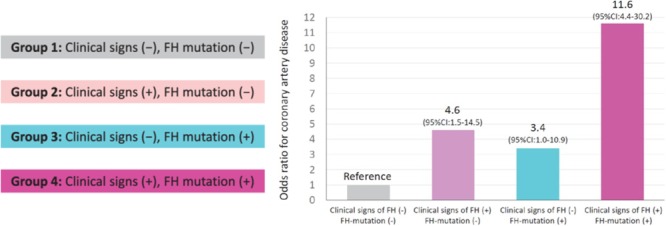
Influence of FH, clinical signs and genetic diagnosis on the risk of CAD. Reprinted with permission from a previous study (Tada et al., 2017). The calculation of odds ratios was performed by logistic regression after adjusting for age, sex, diabetes, hypertension, smoking, and the LDL-C level. FH represents familial hypercholesterolemia; CAD represents coronary artery disease; and CI represents confidence interval (We received copyright permission from Oxford to reproduce the figure).

Screening is recommended to involve both cholesterol testing and genetic analysis (Gidding et al., 2015). When considering only the LDL-C level, approximately 20% of the family members exhibiting LDLR mutations and slightly increased LDL-C level will remain undiagnosed because there is an overlap in LDC-L level between FH and non-FH patients (Umans-Eckenhausen et al., 2001). In addition, classical FH gene (*LDLR, APOB, PCSK9*, and *LDLRAP*) mutations were not detected in 10–40% of patients who met the clinical diagnostic criteria (Nordestgaard et al., 2013). Using genetic testing and evaluation of FH clinical characteristics to identify true FH patients will be very valuable for the stratification of CVD risk. According to the recent retrospective study of a stratified cohort (Tada et al., 2017), patients with any clinical signs or genetic mutations were 11 times more likely to develop CAD than control patients (patients with neither clinical signs nor genetic mutations) after adjusting for any known risk factors, such as smoking and LDL-C level (Figure 4). Screening can help remind younger patients with minor symptoms to strictly control their blood lipid levels (Alonso et al., 2014; Silva et al., 2016) as well as markedly increase the proportion of patients receiving treatment and improve treatment initiation and compliance (Tada et al., 2017). For older subjects, screening can also reveal valuable information for their offspring and facilitate the stratification of CAD risk (Santos et al., 2016).

However, genetic testing also has some disadvantages. Many individuals fear genetic diagnosis for personal reasons, and in less affluent countries, including China, the expense is excluded from medical insurance coverage, and the test has not yet been considered a “standard treatment” (Nordestgaard and Benn, 2017). In China, the cost of whole-exome sequencing is $880 per sample, and the cost of blood lipid-related multigene panels is $300 per sample. Costs for clinical assessments were conservatively estimated as $100 (inclusive of cardiologist, electrocardiogram, biochemical and lipid parameters, and carotid ultrasound) based on Medicare rates. The cost of lifetime clinical screening was estimated for individuals grouped by their ages at the time of genetic testing based on the expected total number of evaluations until the age of 75 years (Gersh et al., 2011). The costs of blood lipid-related genetic analysis will probably be substantially reduced compared with those for regular follow-up clinical assessments, but additional data from China are required.

## Spectrum of Mutations Causing FH in China

Approximately 143 reports of FH focused on Chinese individuals (Accessed Aug 2018). A previous systematic review (Jiang et al., 2015) collected 80 reports about FH-related mutations in Chinese populations and showed that major variants were mapped to three genes, namely, *LDLR, APOB* and *PCSK9*. As expected, among these variants, mutations in the *LDLR* gene (>80%) were most often reported, and 30 of these mutations were considered *novel* variants. China has not yet reported the AR mode associated with the *LDLRAP1* gene. In fact, the adopted diagnostic criteria are different and fail to reflect the diversity of the genetic background of Chinese FH patients, although an increasing number of published articles have begun to pay attention to FH. There is still a large gap between foreign and Chinese studies. Most Chinese studies focused on atherosclerotic manifestations and the representation of genetic code and seldom carried out functional experiments for the investigation of causative mutations.

On the basis of several studies, the *LDLR* gene was most frequently reported. Jiang L (Jiang et al., 2015) and Adzhubei, I. A. (Adzhubei et al., 2010) proved that the distribution and largest percentages of *LDLR* gene mutations lie in exon 4 and in exons 9, 13, and 14, respectively. In addition, the proportions of missense mutations, non-sense mutations and large deletions were 60.3, 13, and 2.3, respectively. Nevertheless, the functions of only 30.5% of these gene mutations were identified (Jiang et al., 2015). Among all *LDLR* gene mutations, a total of three mutations appeared at a high frequency, including the C308Y (c.986G > A, p.Cys329Tyr), the H562Y (c.1747C > T, His583Tyr) and the A606T (c.1879G > A, p.Ala627Thr) variants. The three mutations were present in 23% of probands in China. In addition, southern and northern gene mutation distribution characteristics are also different. The most common ranking of the top 3 mutations and their frequencies in northern China was A606T (18.5%), D601Y (14.8%) and 313 + 1G > A (7.4%). W462X (c.1448G > A, p.Trp483X), A606T (c.1879G > A, p.Ala627Thr), and L393R (c.1241T > G, p.Leu414Arg) are dominant mutations in southern China and were detected in 10.7, 7.5, and 5.4% of the probands, respectively. Furthermore, Jiang et al. (2015) identified 30 mutations not recorded in the abovementioned *LDLR* databases, with missense mutations as the most common mutation type detected in 63.3% of the probands.

As with studies in foreign countries, only a small number of studies in China refer to *ApoB* and *PCSK9* gene mutations (Jiang et al., 2015). R3500W (c.10707 C > T, 50/56) in exon 26 acts as the most common *ApoB* mutation [reported in 1998 by Huang et al. (1988)] and accounts for <10% of FH cases from southeast China (Chiou and Charng, 2012). The *PCSK9* gene mutation R306S was first reported by Lin et al. (2007) (51) and has a low frequency. As identified by Song et al. (2012), 5% of hyperlipidemia patients exhibited *PCSK9* gene mutations. The six *PCSK9* mutations intron 2T > G, R306S, V312S, V312F, R319E, and D320N have already been reported previously (Jiang et al., 2015).

FH is clinically classified into HoFH and HeFH types, and HoFH is rare (Cuchel et al., 2014). China is a multiethnic country with a large population base and has at least 2,000 HoFH patients (Figure 3). However, only approximately 100 HoFH cases were reported in China; thus, the diagnosis of Chinese FH, especially HoFH, and its management have room to improve. Relevant experts recommend that large registry systems for rare diseases should be used to dynamically monitor FH patients and provide early prevention strategies.

## Management of FH

The latest EAS guidelines emphasize that children and adolescents with FH should undergo early screening and management to achieve a longer life (Wiegman et al., 2015). Recent studies (Huijgen et al., 2012a; Nordestgaard et al., 2013) have also proposed a “cumulative LDL-C burden” concept, suggesting that normal 55-year-old individuals with cumulative LDL-C levels of 160 mmol will develop CHD. If untreated, 35-year-old HeFH patients can develop CHD, but if treated at age 18 or 10, the age of CHD onset can be extended to 48 or 53 years old. HoFH patients have a gene accumulation effect that leads to the development of CHD at 12.5 years old if not treated. Transthoracic Doppler ultrasound in HoFH Chinese patients aged 4 to 30 years showed that all patients had significant carotid thickening and aortic plaque burden; 45% of patients had plaque involving a coronary artery opening, and coronary flow reserve was significantly reduced, suggesting that patients have myocardial ischemia at an early age (Yang et al., 2010).

Experts and researchers recommend that children should be screened from the age of 2 and that drug intervention should be initiated at 8 years old for HeFH and no later than 7 years old for HoFH (Thompson, 2015) to maximize the delay in developing CHD and disease progression (Ridker, 2014; Daniels, 2016). The youngest Chinese HoFH patient who underwent drug intervention was 7 years old, and although combination therapy failed to lower the patient’s cholesterol to the target level, early treatment attenuated the formation of atherosclerotic plaques (Jiang et al., 2014). Therefore, early management of FH patients can help achieve a longer life.

Shi et al. (2014) surveyed the largest number of people in China, totaling 9,324 Chinese adults, and revealed that with LDL-C as the clinical diagnostic criteria for FH, the prevalence of FH in Jiangsu Province was 0.31%, while that among people over 50 years old was 0.65%. Chinese experts agreed that the target values of LDL-C in adults with and without ASCVD were <1.8 mmol/L and <2.6 mmol/L, respectively, while the target value of LDL-C in children was <3.4 mmol/L, and secondary conditions reduced serum LDL-C levels by at least 50% (Department of Cardiovascular Diseases, 2018). Although most patients with elevated cholesterol were taking lipid-lowering drugs, almost no patient could achieve the LDL-C target levels recommended by existing diagnostic criteria.

Today, the main treatment options for lowering lipid levels include improving diet and lifestyle and taking medications. However, primary FH usually does not respond to diet, in contrast to secondary hypercholesterolemia. Adult and pediatric FH patients should be recommended to start medications as soon as possible after diagnosis. Statins are first-line drugs for the treatment of FH, and HeFH patients with at least 50% LDLR function show a good response to statins, but HoFH patients respond poorly to these drugs. The treatment efficacy may be related to race, as Chinese patients treated with conventional doses of statins respond poorly, and their tolerance to high-dose statins is generally worse than that of Westerners (Chinese guidelines on prevention and treatment of dyslipidemia in adults, 2016). For patients with multiple comorbidities, concerns of a potentially higher likelihood of adverse drug reactions from high-dose drug regimens often outweigh perceived benefits from uptitration of drug doses (Park et al., 2012). The reason for a patient’s failure to attain the treatment goal might be partly attributed to personal compliance problems, socioeconomic status, education level, occupation, and medical insurance coverage. FH patients should be given, in addition to statin therapy, a lipid-lowering drug, including ezetimibe, cholic acid chelating agents, niacin, and probucol. However, at present, the current clinical application of probucol is not recommended because it lowers HDL cholesterol levels and prolongs the QT interval, and the only drugs approved for marketing in China are statins, ezetimibe, and niacin. Other drugs lack safety and efficacy data for the Chinese population and remain in the research and development stage before the Phase II and Phase III clinical trials in China. In addition, the risk of muscle toxicity was increased when niacin and statins were taken together. As a result, most Chinese FH patients use statins combined with ezetimibe to reduce blood lipid levels. For HoFH children, the safest and most effective treatment is lipid apheresis (Thompson, 2015), but due to the level of compliance in children, the equipment is not frequently used, and the technique is rarely carried out in China. Available data indicated that as a novel therapeutic medicine developed in recent years, PCSK9 inhibitors used with statins plus ezetimibe can better reduce LDL-C levels (Lloyd-Jones et al., 2016). In August 2018, the first PCSK9 inhibitor was approved in China for the treatment of HoFH in adults or adolescents over 12 years of age (World Health Organization, 2018a), thus enabling the development of new approaches to the treatment of patients with FH in China.

There remains a gap in treatment between China and Europe or the United States Fortunately, the relevant departments in China have begun to note the importance of understanding FH and have begun to promote the introduction of Rebene^®^ (eloiubizab evolocumab) injection, which was approved by the China National Drug Administration on July 31, 2018, becoming the first PCSK9 inhibitor for the treatment of HoFH adults or adolescents over 12 years old in China. The approval for Rebene in China is valuable and timely for HoFH patients, bringing them hope for longer life and better quality of life.

## The Need to Establish a Chinese FH Registry System and Genetic Database

Early screening and intervention can effectively delay the progression of atherosclerosis. EAS guidelines show the diagnostic rate of FH in most countries, revealing a diagnostic rate of less than 1% in China as well as in Taiwan and Hong Kong populations, while the data for mainland China are not available (Wiegman et al., 2015). These findings do not suggest that there are no FH patients in mainland China; these data suggest that because the mainland populations do not understand FH, FH patients exhibit a serious deficit in diagnosis and inadequate treatment. This finding prompted us to establish the Chinese FH registration system, which can effectively improve FH awareness in clinicians and reduce the incidence of cardiovascular events in FH patients via preventive measures. The FH genetic database will help to determine the most common gene mutations/variants among Chinese individuals and to identify novel mutations specific to this population, thus contributing to a quick molecular diagnosis and precise medical treatment. At present, many countries in the Netherlands, the United States, Canada, and Australia are establishing an FH registry, with mainly the Netherlands registry serving as the primary model (Umans-Eckenhausen et al., 2001; Bamimore et al., 2015). From the international perspective, the International FH Foundation recently established an extremely tolerant and consensual guidance system that is well designed to manage FH (Watts et al., 2014) and can conveniently promote a patient database at various stages as the first step to launch the full registry systems for more parties. It is noteworthy that because information on the FH treatment program was mostly obtained from foreign research, there is an urgent need for clinical research data from domestic FH patients to enable further development of treatments at the national level. In addition, the safety and tolerance of chronic lipid-lowering drug use in Chinese FH children still require further attention.

The clinical application of genetic databases for precisely diagnosing heart disease relies on a large amount of reliable and accurate clinical guidance data. The registry systems and databases can provide a clearer understanding of biologically significant information, help to integrate data into clinical knowledge with guidance value, and facilitate the use of relevant data by clinical personnel. On the other hand, the process of collecting patient information is still being improved, such that accurate heart disease diagnoses in FH patients using clinical data requires more than information collection at a single time point and emphasizes more regular, continuous clinical records. Therefore, developing a collection strategy to achieve long-term monitoring and analysis largely depends on the attention of clinicians and patients and good medical follow-up. Careful records of FH cases in registry systems and genetic databases will enable improvements in clinical research in China, including scientific collaboration, meta-analysis research, and international clinical trials. Relevant departments are also expected to use the results of the registration system to study the natural history and treatment of FH in Chinese people to provide a basis for diagnosis and more economic treatment.

To this point, the National Rare Disease Registration System in China, led by our team, is designed to establish a large-scale Internet-based registration platform and biobank to publish standardized operating procedures for rare diseases such as HoFH (Feng et al., 2018). The platform connects key infrastructure tools such as databases, registries, biobanks, and clinical bioinformatics. As with the CASCADE FH Registry (O’Brien et al., 2014), the primary source of information will be patient medical records. The baseline data elements entered included patient demographics, FH history and diagnosis, medical history, current lipid-lowering therapies, physical examination findings, and laboratory values. The registration system is designed for cohort studies to facilitate regular entry of follow-up data. This program performed a cohort study of 50,000 cases of at least 59 rare diseases, including FH. Over 20 top academic medical institutions in China are participating in the project. More than 30,000 cases have been recorded in the system since 2017. The current shortcoming of the database is that it cannot be connected with the medical record electronic system of each clinical medical center. All the information needs to be entered by the clinician after logging into the registration system, which virtually increases the non-medical workload of the clinician. However, this platform allows domestic and international collaboration and will provide support for communication between experts, the organization of patient recruitment, data integration and analysis, and patient advocacy for rare diseases worldwide.

FH registry systems and genetic databases are not only clinically important for early screening of FH but are also important for standardized treatment and long-term management. In addition to the conditions of the FH registration system in China and the effective support of genetic databases, public awareness is essential to improve the prognosis of patients with FH. This tool can help develop clinical treatments and determine the most proper practice guidelines for Chinese populations.

## Conclusion

FH is an important risk factor for early onset CVD, and its pathological changes begin in childhood. Therefore, early FH screening and treatment are of great significance for early CVD prevention. The number of FH patients in China is expected to reach 5.5 million, but the diagnosis and treatment rate is less than 1%. Regardless of existing treatment, the clinical target is difficult to achieve in patients with high blood lipids levels, which leads to the occurrence of early cardiovascular events.

**FIGURE 5:**
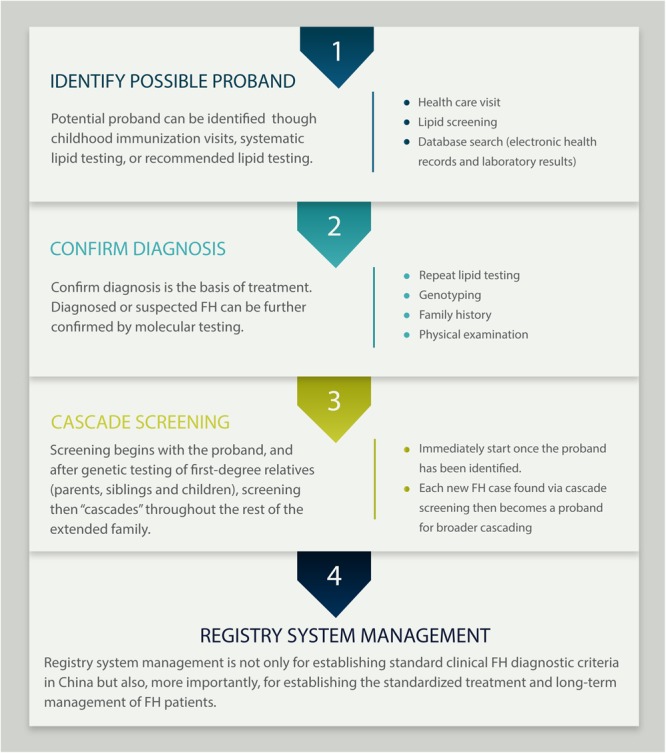
Process flow from case identification to entry in the registry system.

Advocating the establishment of an FH registry system and database is one of the elements of precision medical care and an important rate-limiting step for improving long-term prognosis in FH patients. We recommend a process flow from case identification to entry into the registry system (Figure 5). We can foresee that precise care of FH can further advance the assessment of CVD risk in clinical practice, improve existing diagnostic strategies and expand the methods of CVD intervention, which will further reduce CVD occurrence and provide support for improving prognosis in FH patients. Furthermore, the widespread use of registry systems and genetic databases in clinical applications requires clarification of the potential link between biomechanics and phenotypic information to enhance the capacity of large-scale multidimensional information processing and the ability to translate research results directly into clinical knowledge, thus providing better guidance for medical practice.

## Author Contributions

SYZ conceptualized and designed the study, revised and approved the final manuscript. PPC designed the study, collected, interpreted, and analyzed the data, and drafted the manuscript. XC collected data of the manuscript.

## Conflict of Interest Statement

The authors declare that the research was conducted in the absence of any commercial or financial relationships that could be construed as a potential conflict of interest.

## Footnotes

1 http://www.stats.gov.cn/tjsj/
